# Eight weeks of high-intensity interval training increases peripheral serotonin transporter expression independently of promoter methylation changes

**DOI:** 10.1007/s00421-026-06183-z

**Published:** 2026-03-14

**Authors:** Florian Javelle, Miriam Ringleb, Alexander Schenk, Walter Pulverer, Wilhelm Bloch

**Affiliations:** 1 NeuroPsychoImmunology research unit, Department for Molecular and Cellular Sports Medicine, Institute for Cardiovascular Research and Sports Medicine, German Sport University Cologne, Cologne, Germany; 2 Department of Movement Science, University of Münster, Münster, Germany; 3 Department of Sports Medicine and Health Promotion, Friedrich-Schiller-University Jena, Jena, Germany; 4 Sports Medicine Research Group, Institute for Sport and Sport Science, TU Dortmund University, Dortmund, Germany; 5 Molecular Diagnostics, AIT Austrian Institute of Technology GmbH, Vienna, Austria; 6 Department for Molecular and Cellular Sports Medicine, Institute for Cardiovascular Research and Sports Medicine, German Sport University Cologne, Cologne, Germany

**Keywords:** Serotonin transporter, 5-HTT, Gene expression, Methylation, MAO-A, Monoamine oxidase A, Exercise, High-intensity interval training

## Abstract

**Purpose:**

Impulsivity, a personality construct linked to various psychiatric disorders, is partially regulated by serotonergic neurotransmission. While genetic polymorphisms affecting serotonin transporter (5-HTT) transcription have been widely studied, the role of epigenetic modifications, particularly DNA methylation, remains unclear. Building on earlier analyses from the present cohort that demonstrated that eight weeks of physical exercise reduces impulsivity, this study investigated whether these behavioural changes are accompanied by alterations in 5-HTT expression together with 5-HTT and MAO-A promoter methylation in buffy coat (BC) cells.

**Registry:**

drks.de, TRN: DRKS00016589, Registration date: 6 February 2019.

**Methods:**

Participants (*n* = 45) were randomly assigned to either high-intensity interval training (HIIT) or stretching (active control). Each group completed three training sessions per week for eight weeks, with pre- and post-intervention assessments of impulsivity, 5-HTT gene expression, and 5-HTT and MAO-A promoter methylation.

**Results:**

Results indicate a significant increase in 5-HTT expression in the HIIT group compared to controls (*p* = .008, *η*_*p*_^*²*^ = 0.156; *d* = 0.782), but no corresponding changes in promoter DNA methylation. Moreover, changes in 5-HTT expression did not correlate with changes in impulsivity. Specific 5-HTT and MAO-A promoter methylation changes were weakly associated with certain impulsivity factors.

**Conclusion:**

These findings suggest that exercise influences serotonergic function by increasing 5-HTT expression independently of promoter DNA methylation changes. Further research is needed to determine whether these changes are also present at central nervous system level or if this upregulation is primarily a result of long-term anti-inflammatory effects triggered by physical exercise.

**Supplementary Information:**

The online version contains supplementary material available at 10.1007/s00421-026-06183-z.

## Introduction

Impulsivity is a multidimensional personality construct linked to various psychological disorders (Corruble et al. 1999; Swann et al. 2001; Carver et al. 2008; Johnson et al. 2017; Javelle et al. 2023). While early definitions of impulsivity focused on problems with planning, deliberation, and attention (Barratt 1965; Dickman 1990), more recent research highlights the clinical relevance of impulsivity in response to intense emotions (Whiteside and Lynam 2001; Carver et al. 2008, 2011). Indeed, the latter has shown stronger associations with psychopathologies, aggression, and suicide than other forms of impulsivity (Carver et al. 2008; Smith and Cyders 2016; Johnson et al. 2017).

Serotonin (5-HT) is one of the key neurotransmitters involved in regulating impulsivity and emotion (Dalley et al. 2008; Tricklebank and Daly 2018). The serotonin transporter (5-HTT), which mediates 5-HT reuptake from the synaptic cleft, plays a crucial role in serotonergic neurotransmission (Canli and Lesch 2007; Tricklebank and Daly 2018). While mostly known for its central effects, 5-HTT is also expressed in platelets and certain immune cells (e.g., lymphocytes, monocytes/macrophages) in the periphery (Herr et al. 2017). 5-HTT is encoded by the SLC6A4 gene, whose transcription is influenced by genetic variations, including the serotonin transporter-linked polymorphic region (5-HTTLPR), a single-nucleotide polymorphism (rs25531), and the serotonin transporter intronic region 2 (STin2) (Canli and Lesch 2007; Iurescia et al. 2017). Research investigating the relationship between these polymorphisms and impulsivity-related disorders suggests that individuals with genetic variants linked to reduced 5-HTT transcriptional activity (5-HTT_Low_) may have a higher prevalence of such disorders (Canli and Lesch 2007; Clarke et al. 2010; Carver et al. 2011; Kenna et al. 2012; Pavlov et al. 2012). In a recent study, we confirmed the existence of this association in healthy humans, showing that individuals with genetic variants linked to 5-HTT_Low_ exhibit greater emotion-related impulsivity (ERI; behavioural form) compared to others (Javelle et al. 2022a). However, the strength of the association between impulsivity-related disorders and 5-HTT genetic variants varies across studies, potentially indicating the involvement of other important genes for serotonergic neurotransmission, like the monoamine oxidase A (MAO-A), which catabolises monoamines (Javelle et al. 2022a), and/or additional mechanisms, such as epigenetic regulations.

Unlike genetic modifications, epigenetic changes are reversible and do not alter DNA sequences, yet they can influence how genes are read (Jaenisch and Bird 2003). One of the most studied epigenetic mechanisms is DNA methylation, which involves the addition of a methyl group (CH3) to cytosine within cytosine-guanine dinucleotides (CpG) (Jaenisch and Bird 2003; Kim and Costello 2017). Proper methylation patterns ensure that genes are activated or silenced at the right times, which is essential for embryonic development, immune function, and neurological processes (Li et al. 1992; Okano et al. 1999). Typically, methylation-mediated gene regulation occurs at gene promoters, which often contain cytosine-rich regions known as CpG islands (Jaenisch and Bird 2003; Kim and Costello 2017). For example, the 5-HTT gene features a 799 bp CpG island with 74 CpG sites, making it a key target for methylation studies in neuropsychiatric research.

When methylation becomes dysregulated, it can lead to severe health consequences. In neurological and psychiatric disorders, altered methylation patterns in key genes involved in 5-HT neurotransmission, such as the 5-HTT, have been associated with impulsivity-related disorders (Dukal et al. 2015; Duman and Canli 2015; Zhu et al. 2019; Ikegame et al. 2020; Akhrif et al. 2023). However, in our latest meta-analysis on depressive disorders, we demonstrate that this relationship is not evident, and findings remain heterogeneous (Javelle et al. 2025). This heterogeneity may stem from the complexity of methylation processes. Methylation is cell-specific, meaning that increased methylation in peripheral tissues does not necessarily reflect increased methylation in the central nervous system (CNS), particularly in brain structures where 5-HTT is most likely to influence behaviour. However, routinely collecting CNS biological samples from humans without creating any inconvenience still remains a significant challenge and is not likely to be possible soon. Therefore, while indirect, a better understanding of the relationship between peripheral and central methylation levels and how they relate to impulsivity and IR disorders could provide valuable insights.

Several studies have demonstrated that promoter methylation and genetic polymorphisms interact, potentially explaining some of the heterogeneity observed in earlier research that assessed them separately (Olsson et al. 2010; IJzendoorn et al. 2010; Kinnally et al. 2011; Drabe et al. 2017; Lam et al. 2018; Ikegame et al. 2020). Considering both promoter methylation and polymorphic region in serotonergic neurotransmission genes could help explain why some individuals with a high-transcriptional activity 5-HTTLPR phenotype still exhibit low 5-HTT mRNA levels. This might also clarify inconsistent findings regarding the association between 5-HTTLPR polymorphism and impulsivity-related disorders. However, this hypothesis remains to be fully tested, together with 5-HTT expression levels.

While the genome is unchangeable, humans can regulate their methylation levels through healthy lifestyle habits, including regular physical exercise, a healthy diet, and proper sleep (Elgendy et al. 2018; Lahtinen et al. 2019; Fernandez-Sanlés et al. 2021). Functional genomic studies on DNA methylation have demonstrated the impact of lifestyle interventions (e.g., exercise or diet) on health and behaviour (Elgendy et al. 2018; Fernandez-Sanlés et al. 2021). For instance, Fernandez-Sanlés et al. (2021) identified an association between physical activity and methylation at specific CpG sites. However, no studies to date have investigated how exercise influences methylation in serotonergic genes and its subsequent effects on gene expression. In a previous intervention study, we demonstrated that eight weeks of high-intensity interval training (HIIT) reduced both non-emotion-related impulsivity (nERI) and ERI levels in highly impulsive individuals (Javelle et al. 2021). This raises the question of whether these changes are driven by semi-functional modifications detectable in the peripheral cells, specifically, methylation changes that regulate serotonergic gene expression.

Building on data from the NoSTRESS study (which identified a link between 5-HTT polymorphisms and impulsivity, as well as a reduction in impulsivity through physical exercise), this exploratory project aims to investigate whether 5-HTT promoter methylation and expression in buffy coat (BC) cells, together with the tri-allelic 5-HTTLPR polymorphism, can further explain variations in impulsivity levels. We first examined whether there is an association between BC 5-HTT methylation and expression levels. Next, given that the exercise interventions in NoSTRESS led to decreased impulsivity levels, we assessed whether the eight-week exercise program influenced peripheral 5-HTT expression and contributed to changes in DNA methylation. Finally, we determined whether changes in these molecular markers correlate with variations in impulsivity. We hypothesised that methylation within the 5-HTT promoter region, together with its polymorphism, would better account for variability in impulsivity levels. Additionally, we expected that exercise would induce changes in both 5-HTT expression and methylation. As impulsivity has also been associated with genetic variants of MAO-A (Williams et al. 2009; Javelle et al. 2022a), and since catecholamine regulation by physical exercise has already been demonstrated (Zouhal et al. 2008), changes in methylation of the MAO-A promoter region were also explored. However, because MAO-A is poorly or not expressed in BC at rest, its gene expression was not assessed.

## Methods

This study is a secondary analysis of the NoSTRESS project (Javelle et al. 2021, 2022a, b, 2024). Planning and implementation of that project were carried out following the Declaration of Helsinki and the Guidelines for Good Clinical Practice. The NoSTRESS study was registered on the German clinical trial registration website (DRKS00016589) and approved by the university review board (No. 166/2018).

### Experimental design

Individuals with high impulsivity levels and a sedentary lifestyle, assessed via an online screening questionnaire, were enrolled in the study. This online assessment included a brief evaluation of ERI using the Feeling Trigger Action scale (Javelle et al. 2020), along with questions addressing inclusion and exclusion criteria. Participants were then randomly assigned to an eight-week intervention, consisting of either HIIT or light stretching, performed three times per week.

All exercise sessions were conducted under supervision at the German Sport University Cologne. Each session lasted 35 min, including warm-up and cool-down periods. Exercise intensity was adjusted to individual capacity based on baseline (T0) cardiopulmonary exercise testing heart rate results. For further details on the screening procedure and the initial in-person meeting, refer to (Javelle et al. 2022a, b). Additional information on the exercise intervention and primary aims of the NoSTRESS project can be found in (Javelle et al. 2021, 2024).

This secondary analysis focuses on blood samples and impulsivity assessment collected before (T0) and after (T8) the eight weeks of exercise intervention.

### Sample

A total of 45 participants completed the intervention (26 in the HIIT group and 19 in the stretching group), each attending at least 20 out of the 24 required training sessions. Participants from the HITT group were mostly females (57.7%), with an average age of 31.2 years (± 8.2 years) and a BMI of 24.2 ± 3.5 kg/m^2^. Participants from the stretching group were also mostly females (73.7%), with an average age of 29.1 years (± 8.1 years) and a BMI of 25.6 ± 3.8 kg/m^2^ (Javelle et al. 2024).

### DNA and RNA isolation

Before (T0) and after (T8) the intervention period, 17 mL blood samples were collected into K2 EDTA tubes from fasted participants in the morning. BC (containing peripheral blood mononuclear cells, granulocytes, and platelets) was isolated through two rounds of alternating centrifugation and phosphate-buffered saline (PBS) resuspension. A freezing medium was then added at a ratio of 1 mL per 300 µL of BC, and samples were stored at −150 °C.

On the day of DNA and RNA isolation, the BC was thawed and centrifuged for 10 min at 5500 rpm. After discarding the supernatant, the pellet was resuspended in 200 µL of PBS. DNA was extracted using the Blood DNA Mini Kit (Bio-Budget Technologies GmbH, Germany) and stored at −20 °C until analysis. For gene expression assessment, RNA was isolated using a column-based kit (T2010 Monarch Total RNA Miniprep Kit, New England BioLabs GmbH, Germany) and stored at −80 °C until analysis.

Quantitative and qualitative assessments of DNA and RNA were performed using the NanoDrop 1000 spectrophotometer (Peqlab Biotechnology GmbH). Purity was determined by measuring the A260/A280 and A260/A230 absorbance ratios under UV light (Gallagher and Desjardins 2006). Purity ratios were between 1.4 and 2.2 for DNA and between 1.5 and 2.5 for RNA (A260/A280), while the A260/A230 ratios were between 1.5 and 2.4 for both.

Subsequent 5-HTT and MAO-A polymorphism analyses were conducted on the extracted DNA, as detailed in Javelle et al. 2022a, 2022b.

### Methylation

#### Methylation-sensitive restriction enzymes analysis

The DNA samples were sent to the Austrian Institute of Technology in Vienna, where methylation was assessed through methylation-sensitive restriction enzymes (MSRE). Using this method, 22 different assays were designed for the 5-HTT gene (Fig. A, Supplementary Material A), covering a 945 bp region (Assembly GRCh38/Hg38, chr17: 30,235,304 − 30,236,249). This region includes a known 799 bp CpG island and targets 74 individual CpG sites within the promoter region. For the MAO-A gene, 16 assays were developed (Fig. B, Supplementary Material A) covering two CpG islands (Assembly GRCh38/Hg38, chrX: 43,656,003–43,656,472 and chrX: 43,654,686 − 43,655,616) targeting 12 and 40 distinct CpG sites within the promoter region, respectively. The exact genomic coordinates of each CpG site analysed in each assay are detailed in Supplementary Material A.

Two methods were used for DNA methylation analysis: a microfluidic high-throughput quantitative PCR (qPCR) (µHT-qPCR) readout with the Biomark system (Standard Biotools Biomark, San Francisco, USA) and a conventional readout on a Roche LightCycler with a 384-well plate. In principle, the workflow was identical for both readout methods, and those assays that did not yield satisfactory results with the Biomark system were analysed with the Roche LightCycler. In brief, DNA was digested with a combination of four different MSRE (HpaII, Hin6I, AciI, HpyCH4IV) in Tango buffer (Thermo Fisher). The assays for the targeted regions were designed using Primer3 software, ensuring that at least two cut sites for the MSREs were located in the targeted region. The DNA methylation readout was performed with MSRE-digested and undigested DNA to normalise the DNA input. A more detailed description of the process, including the reagents used, can be found in Supplementary Material A.

The qPCR efficiency per assay was tested by serial dilution. Based on these results, five assays for 5-HTT and four for MAO-A were excluded due to inadequate efficiency (Supplementary Material A). Therefore, 17 assays covering 64 CpG sites for 5-HTT and 12 assays covering 43 CpG sites for MAO-A were included in the further analysis.

#### Data analysis

In the initial step, the raw data were filtered based on melting temperature (Tm). Samples with deviating Tm values of more than +/- 1.5 °C from the reference Tm value (median of all measured Tm values over an assay) were excluded from further analysis. Then, the delta cycle threshold (∆Ct) values between the digested reaction and the undigested reaction of the sample were calculated. For digested reactions, in cases where DNA is completely digested, resulting in 100% unmethylation, no qPCR signal is detected. Consequently, a Ct value of 45, representing the maximum number of qPCR cycles, was imputed. For undigested reactions that failed to produce a Ct value, the sample was deemed unsuccessful and excluded from further analysis in that assay. Finally, to assess changes across the intervention, values obtained at T8 were subtracted from those gathered at T0. Therefore, a positive final outcome refers to an increase in methylation, while a negative one refers to a decrease in methylation.

### Gene expression

The assessment of 5-HTT expression was performed following the MIQE guidelines (Bustin et al. 2009) using two-step reverse transcription qPCR (RTqPCR). Actin beta (ACTB) and Ribosomal Protein S18 (RPS18) were selected as reference genes. Primers were designed using Primer-BLAST, and their annealing temperatures were verified through gradient PCR and gel electrophoresis. Primer efficiency was assessed using a tenfold serial dilution. Multiple genes and qPCR protocols were tested on pilot samples until all primers achieved an efficiency between 95% and 105%. The validated primers and RTqPCR conditions are detailed in Table 1. qScript cDNA synthesis kit (Quanta Biosciences, Inc., United States) was used for the reverse transcription, and Perfecta SYBR green fast mix (Quanta Biosciences, Inc., United States) was used for the qPCR.

**Table 1:** Information about primers

Gene	Primer sequence	AT	Efficiency	RTqPCR Protocol	
5-HTT	Fwd: ACAAAGTGGAGTCCGGGCAA Rev: GTAGGGGAAGCGCCAGACAT	63 °C	99.1%	2 *min* at 95 °C	
				10 *sec* at 95 °C	40 cycles
				45 *sec* at 62 °C	
ACTB	Fwd: ACAGA GCCTC GCCTT TGCC Rev: GCGCG GCGAT ATCAT CATCC	63 °C	101.4%	45 *sec* at 72 °C	
				1 *min* at 95 °C	
RPS18	Fwd: ATTAA GGGTG TGGGC CGAAG Rev: TGGCT AGGAC CTGGC TGTAT	60 °C	102.0%	45 *sec* at 62 °C	
				30 *sec* at 95 °C	

*5-HTT* serotonin transporter, *ACTB* actin-beta, *AT* annealing temperature, *Fwd* forward, *min* minures, *sec* seconds

Since genomic DNA (gDNA) contamination was detected in the study samples, RNA samples were successfully treated with DNase I (Promega GmbH) to remove any remaining gDNA before proceeding to the analysis. Non-target controls (NTCs) and minus reverse transcriptase (MRT) controls were included in the analysis to control for contamination.

Finally, the 5-HTT expression was computed using the relative efficiency-corrected comparative quantification method according to Pfaffl’s formula (Pfaffl 2001). The relative comparison between T0 and T8 was determined with the values for 5-HTT normalised to ACTB and RPS18, which were then averaged. Since T8 is subtracted from T0, a value of 1 indicates no change from T0 to T8, while a value greater than 1 represents an increase in expression levels, and a value smaller than 1 indicates a decrease.

For six participants, values for the relative comparison had to be interpolated due to a missing time point or determination problems occurring during the RTqPCR. Their interpolation was based on the average value of the relative comparison of their corresponding intervention group. However, when considering expression only at T0, no imputation was performed, as it was deemed less reliable.

### Three-factor impulsivity index (Carver et al. 2011)

The Three-Factor Impulsivity Index is a self-report questionnaire consisting of 54 items designed to assess impulsivity. Participants rate each item on a scale from 1 (“Strongly disagree”) to 5 (“Strongly agree”), with higher scores indicating greater impulsivity. The questionnaire measures eight distinct aspects of impulsivity, which, based on oblique factor analysis and confirmatory structural equation modelling, cluster into three main factors (Carver et al. 2011; Javelle et al. 2020): Pervasive Influence of Feelings, Lack of Follow-Through, and Feelings Trigger Action (Cronbach’s alphas: 0.887, 0.901, and 0.878, respectively). Pervasive Influence of Feelings refers to poor constraint over motivation, while Feelings Trigger Action refers to poor constraint over behaviour. Lack of Follow-Through covers issues such as lack of perseverance and distractibility without reference to emotion. The German-validated version of the questionnaire was used (Javelle et al. 2020). Two catch items (“Please select I agree”) were embedded in the Three-Factor Impulsivity index. All participants answered the catch items properly, and no data were removed for failing these items.

### Statistics

Statistical analyses were performed using JASP (v0.18.3). The trial was analysed following per-protocol standards. All variables were examined for linearity using quantile-quantile plots and histograms of standardised residuals, as well as for skewness and kurtosis. A logarithmic transformation was applied to 5-HTT expression values to meet normality and further test assumptions.

Differences in impulsivity across 5-HTT polymorphisms were assessed using gender-controlled ANCOVAs. Differences in 5-HTT expression and in 5-HTT assays methylation across 5-HTT polymorphisms were computed using ANOVAs. Correlations between baseline impulsivity and 5-HTT expression were analysed using Pearson’s correlations. Methylation levels and their associations with both expression and impulsivity were evaluated using Spearman’s correlations.

The effect of the intervention on impulsivity levels was examined through gender and baseline-controlled repeated-measures ANCOVA. Changes in 5-HTT expression, 5-HTT methylation and MAO-A methylation were assessed using gender-controlled ANCOVAs.

## Results

### Association between MAO-A and 5-HTT polymorphisms, expression, promoter methylation and impulsivity at baseline

This analysis focuses on a subsample (*n* = 45) from the dataset previously reported in Javelle et al. (2022a) (*n* = 67). To assess the stability of the original findings, the association between impulsivity and polymorphism categories was reanalysed. Consistent with Javelle et al. (2022a) individuals with the 5-HTTLPR_Low_ phenotype exhibited higher Feelings Trigger Action scores than others (*n* = 45, *η*_*p*_*²* = 0.155, *p* =.032, Fig. C, Supplementary Material A). No significant differences were observed for the other impulsivity factors or for comparison involving MAO-A phenotypes.

Baseline 5-HTT expression (T0) did not significantly differ between 5-HTT polymorphic groups (*n* = 39, *η*_*p*_*²* = 0.019, *p* =.714). Similarly, 15 of the 17 5-HTT methylation assays showed no difference between phenotypes, whereas the remaining two assays revealed significant differences (assay 7, *n* = 44, *p*=.028,* log*_*2*_*(5-HTTLPR*_*Low*_*/5-HTTLPR*_*Moderate*_*)=−0.50*,* log*_*2*_*(5-HTTLPR*_*Low*_*/5-HTTLPR*_*High*_*) = 0.55;* assay 13, *n* = 45, *p*=.006,* log*_*2*_*(5-HTTLPR*_*Low*_*/5-HTTLPR*_*Moderate*_*) = 0.07*,* log*_*2*_*(5-HTTLPR*_*Low*_*/5-HTTLPR*_*High*_*) = 0.84;* Table A, Supplementary Material A). For MAO-A, two assays out of 12 were differently methylated between polymorphic groups (assay 26, *n* = 45, *p*=.025,* log*_*2*_*(MAO-A*_*Low*_*/MAO-A*_*High*_*)=−1.35;* assay 37, *n* = 43, *p*=.021,* log*_*2*_*(MAO-A*_*Low*_*/MAO-A*_*High*_*)=−0.90;* Table B, Supplementary Material A). No significant associations were found between baseline 5-HTT expression and any of the impulsivity factors. Similarly, baseline 5-HTT promoter methylation levels showed no significant correlations with impulsivity factors (Fig. F, Supplementary Material A). However, methylation on assay 2 was positively correlated with baseline 5-HTT expression (*n* = 38, *r*=.401, *p*=.013 - Fig. F, Supplementary Material A). In contrast, several MAO-A promoter methylation assays showed significant associations with impulsivity factors. Methylation at assay 24 was positively correlated with Feelings Trigger Action (*n* = 45, *r*=.298, *p*=.047). Additionally, methylation at assays 31 (*n* = 41, *r*=.322, *p*=.040), 33 (*n* = 45, *r*=.346, *p*=.020), and 36 (*n* = 44, *r*=.348, *p*=.020) was positively associated with Lack of Follow-Through (Fig. G, Supplementary Material A). Overall, methylation levels across assays were positively correlated with one another, particularly within the MAO-A gene (Fig. F and G, Supplementary Material A).

### Effect of the exercise intervention on impulsivity, 5-HTT expression, and methylation

In Javelle et al. (2021), we reported that both intervention groups showed a significant and moderate reduction in ERI between T0 and T8 weeks (Javelle et al. 2021). For the nERI factor, a small to moderate interaction effect was observed, where only the HIIT group showed a decrease between T0 and T8 weeks, while stretching did not change (Javelle et al. 2021). Since the original analysis included three time points, a modified intention-to-treat approach, and mixed models, we reanalysed these data using only T0 and T8 weeks with a per-protocol approach while still adjusting for baseline values and including sex as a covariate. As for the previous analysis, Lack of Follow-Through had a significant time effect (*n* = 45, *η*_*p*_*²* = 0.101, *p*=.035) and a significant interaction (*n* = 45, *η*_*p*_*²* = 0.135, *p* =.014), with a decrease in the HIIT group from T0 to T8 and a significant difference between the HIIT and stretching groups at T8. Feelings Trigger Action still had a large, significant time effect (*n* = 45, *η*_*p*_*²* = 0.228, *p*=.001); nonetheless, Pervasive Influence of Feelings lost it (*n* = 45, *η*_*p*_*²* = 0.013, *p* =.467).

**Fig. 1:**
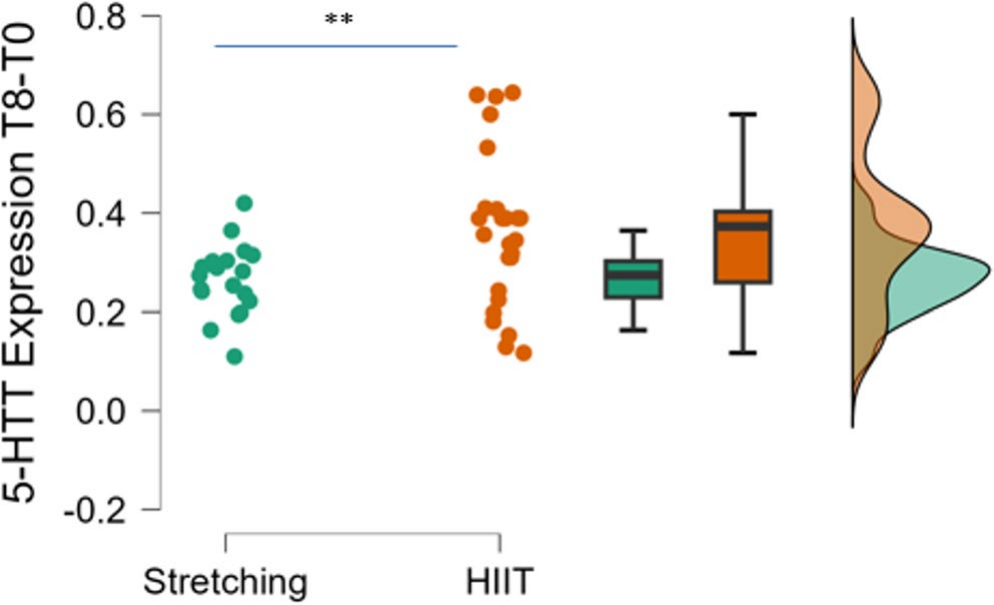
Serotonin transporter (5-HTT) expression from T0 (baseline) to T8 (after 8 weeks of intervention) between stretching and high-intensity interval training (HIIT) groups. * : *p*<.050; **: *p*<.010; ***: *p*<.001

**Fig. 2:**
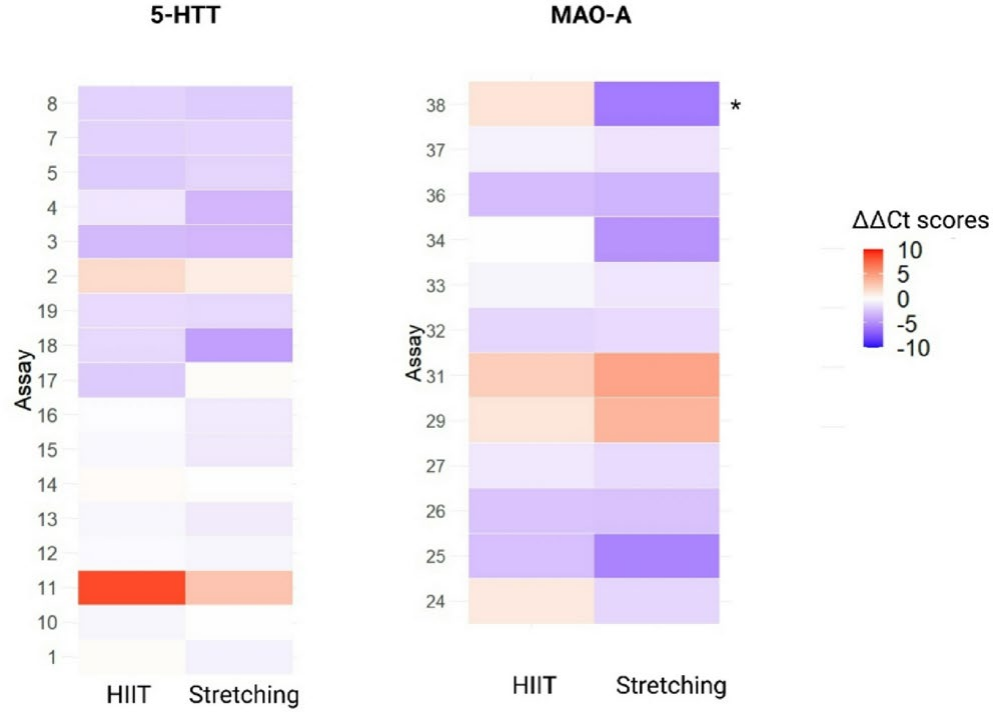
Delta-delta Ct (∆∆Ct) methylation scores from T0 (baseline) to T8 (after 8 weeks of intervention) between stretching and high-intensity interval training (HIIT) groups for the Serotonin transporter (5-HTT) and Monoamine oxidase A (MAO-A) assays. Group differences: * : *p*<.050; **: *p*<.010; ***: *p*<.001

The 5-HTT expression levels between T0 and T8 were significantly different between HIIT and stretching groups (*n* = 45, *η*_*p*_*²* = 0.156; *p*=.008, d = 0.782, Fig. 1). No change in 5-HTT methylation levels was detected (Fig. 2 and Table B, Supplementary material A). Methylation levels at assay 38 of the MAO-A gene were significantly decreased between T0 and T8 in the stretching group compared to the HIIT group (assay 38, *n* = 45, *p*=.018, Fig. 2 and Table D, Supplementary Material A).

### Association of the changes

Changes in 5-HTT expression from T0 to T8 were not associated with variations in Pervasive Influence of Feelings (*n* = 39, *r* = −.069, *p* =.651), Lack of Follow-Through (*n* = 39, *r* = −.106, *p* =.487), or Feelings Trigger Action (*n* = 39, *r* =.052, *p* =.734). Changes in 5-HTT methylation levels were not associated with changes in 5-HTT expression. Changes in 5-HTT methylation levels for assay 4 and assay 7 were positively associated with changes in Feelings Trigger Action (*n* = 44, *r* =.313, *p* =.039 and *n* = 44, *r* =.355, *p* =.018, respectively). Similarly, changes in 5-HTT methylation levels for assay 14 were positively associated with changes in the Pervasive Influence of Feelings (Fig. F, Supplementary Material A). Changes in MAO-A methylation levels for assay 27 and assay 31 were positively associated with changes in Lack of Follow-Through (*n* = 45, *r* =.338, *p* =.023 and *n* = 45, *r* =.371, *p* =.018, respectively). Overall, methylation levels across assays were positively correlated with one another (Fig. I and J, Supplementary Material A).

## Discussion

Building on data from the NoSTRESS study (which established a link between 5-HTT polymorphisms and impulsivity, as well as a reduction in impulsivity through physical exercise), this study investigated whether 5-HTT methylation and expression in BC, together with the tri-allelic 5-HTTLPR polymorphism, could further explain impulsivity differences. It also examined whether reductions in impulsivity following an eight-week HIIT or stretching (active controls) program were accompanied by changes in these molecular markers. Results indicate a significant large increase in 5-HTT expression in the HIIT group compared to active controls (*η*_*p*_^*²*^ = 0.156, *p*=.008), but no corresponding changes in DNA methylation. Moreover, changes in 5-HTT expression did not correlate with impulsivity reductions, although specific methylation changes were weakly associated with certain impulsivity subcomponents. All of those analyses (except for expression) were also tested for the MAO-A promoter methylation in an explorative manner. Several MAO-A promoter methylation assays showed significant associations with impulsivity factors (assay 24 for Feelings Trigger Actions and assays 31, 33, 36 for Lack of Follow-Through). MAO-A methylation changes differed between groups and were linked to impulsivity: assay 38 decreased in the stretching group, and changes at assays 27 and 31 were positively associated with changes in Lack of Follow-Through.

The lack of association between 5-HTT expression and impulsivity levels, both at baseline and in response to the intervention, along with the absence of association between 5-HTT expression and 5-HTTLR polymorphisms, raises critical questions regarding the relationship between peripheral serotonergic markers and CNS function. Notably, part of those findings was also reported by Iga et al. (2016), who found no association between 5-HTT expression and 5-HTTLPR genotype in either depressive patients or controls. One plausible explanation is that 5-HTT expression in BC does not accurately reflect central serotonergic activity and thus is challenging to associate more directly with behavioural regulation. While research suggests that some peripheral biomarkers can serve as proxies for CNS conditions (Janigro et al. 2021; Skorobogatov et al. 2021), those direct associations remain inconsistent (Skorobogatov et al. 2021), particularly for markers like 5-HT that can have distinct roles in peripheral and central contexts.

This discrepancy may arise from the fact that 5-HT is not exclusively synthesised and utilised in the CNS; peripheral production of 5-HT is well-documented (Zimmer et al. 2017; Herr et al. 2017). For instance, approximately 95% of the body’s 5-HT is produced in the gastrointestinal tract by enterochromaffin cells, serving various peripheral functions (Gershon 2013). In the bloodstream, 5-HT is primarily stored in platelets, which uptake 5-HT via the 5-HTT and release it upon activation at inflammatory sites through interaction with soluble factors, platelet-endothelial interactions, bacteria, and parasites (Mössner and Lesch 1998; Wagner and Frenette 2008; Mcmorran et al. 2009). The released 5-HT acts as a pro-inflammatory activator, stimulating monocytes/macrophages, dendritic cells, granulocytes, and lymphocytes (mostly T cells) (Herr et al. 2017). Those cells can express 5-HT receptors from multiple classes (5-HT1, 5-HT2, 5-HT3, 5-HT4, and 5-HT7), the 5-HTT, and key enzymes involved in 5-HT metabolism (Herr et al. 2017). While platelets primarily use 5-HTT to uptake and store 5-HT, other immune cells use 5-HTT to internalise 5-HT, which is then metabolised into 5-hydroxyindoleacetic acid (5-HIAA) (Herr et al. 2017) by MAO-A. This helps to prevent excessive 5-HT accumulation, which could otherwise lead to a dysregulated immune system.

Our findings introduce a new perspective on the relationship between physical exercise and serotonergic function by demonstrating significant changes in 5-HTT expression. However, rather than reflecting direct effects on central serotonergic pathways, the observed increase in peripheral 5-HTT expression following eight weeks of HIIT might be more closely linked to peripheral immune responses triggered by exercise. Indeed, the anti-inflammatory effects of physical exercise have been widely highlighted (Gleeson et al. 2011). This was also observed in the present study as indexed by the reduction of resting IL-6 in the HIIT group (Javelle et al. 2021). This downregulation of pro-inflammatory signals might be associated with a decrease in macrophages, neutrophils and effector T-cells. However, despite their reduced numbers, these cells may exhibit increased 5-HTT expression to suppress pro-inflammatory signalling and promote the anti-inflammatory environment induced by exercise. This can also be associated with a shift in the BC composition, still expressing 5-HTT while being more oriented toward an anti-inflammatory response. Therefore, the increased expression of 5-HTT in BC following HIIT may reflect an adaptive immune response rather than a mechanism directly influencing impulsivity-related behaviours. However, by acting as an immune mediator helping to profile the exercise-induced anti-inflammatory environment, this peripheral upregulation could also influence other biological pathways. In particular, it may contribute to shifts in the peripheral and central balances between neuroprotective and inflammation-driven neurotoxic branches of the kynurenine pathway, thereby indirectly modulating impulsivity. Future research should explore whether this peripheral upregulation of 5-HTT contributes to broader systemic changes, including potential feedback effects on central serotonin and kynurenine metabolism, or whether it primarily serves to fine-tune immune function in response to physical exercise.

During acute exercise, the surge in catecholamines (especially epinephrine) promotes the rapid redistribution of immune cells (particularly NK cells, CD8 + T cells, and monocytes) into the bloodstream, enhancing immune surveillance (Nagatomi et al. 2000; Rumpf et al. 2021). In parallel, catecholamines modulate immune function by suppressing pro-inflammatory cytokine production and promoting anti-inflammatory signalling, contributing to a transient, exercise-induced immunoregulatory state (Nagatomi et al. 2000). Long-term adaptations include reduced baseline catecholamine levels (Cooksey et al. 1978; Winder et al. 1979) and a faster return to baseline following exercise (Purdon et al. 1993). Those points suggest that physical exercise may influence peripheral MAO-A levels, potentially through epigenetic mechanisms. Although assay 38 revealed a slight increase in methylation in the HIIT group and a decrease in the stretching group, no consistent or systemic changes were observed across assays. Future studies should assess MAO-A methylation pre- and post-exercise in the context of chronic interventions, focusing on specific cell types within the BC that express MAO-A and play key roles in 5-HT and catecholamine-mediated immune regulation (e.g., macrophages, neutrophils, dendritic cells).

The associations between 5-HTT promoter methylation levels in BC, impulsivity, and 5-HTT expression were not evident in this study. While baseline methylation at assay 2 (indexing two CpG located at chr17:30,235,765 and chr17:30,235,768) for 5-HTT was moderately associated with 5-HTT expression and changes in methylation at three assay sites showed small to moderate associations with changes in impulsivity, no consistent or systematic patterns emerged. A similar lack of systematic associations was observed for MAO-A methylation. Furthermore, if adjusting for multiple testing (0.05/17 = 0.003 and 0.05/12=0.004), none of these associations remain significant. In our recent meta-analysis exploring the relationship between 5-HTT promoter region methylation levels and depression occurrence and severity, no significant main effects were found in either case (Javelle et al. 2025). Depression is characterised by heightened ERI, particularly in terms of poor constraint over motivation and thought (Pervasive Influence of Feelings) (Johnson et al. 2013, 2017). Since ERI is often more pronounced in individuals with internalising disorders than in our sample, the lack of significant results in this study is not entirely unexpected.

Despite being the first study to consider both methylation and expression levels of 5-HTT together with impulsivity levels, this analysis has several limitations. Given its preliminary nature, single-CpG site analyses (which can be significantly more cost-intensive than MSRE methods) were not employed. Consequently, it is possible that important changes at specific CpG sites with functional relevance were overlooked and diluted when captured in one assay. Future studies should therefore employ higher-resolution methods (e.g., targeted bisulfite sequencing or pyrosequencing) to better capture site-specific epigenetic effects. Additionally, methylation patterns vary between cell types (Hohos et al. 2018; Ebrahimi et al. 2021; Nishitani et al. 2023), and thus methylation in neural cells involved in 5-HT central synthesis or triggering impulsive behaviours, for example, in the raphe nuclei or prefrontal cortex, may have been more relevant. However, obtaining neural cell samples from healthy individuals is highly invasive, making the use of peripheral material as a proxy a reasonable idea, even though it is inconclusive in our case. Finally, although less likely, it cannot be excluded that exercise-induced changes in 5-HTT expression are associated with methylation alterations on the few CpG sites located outside the 799-bp CpG island (e.g., within upstream regions such as the AluJb fragment) which were not captured by the present assays.

This study was also the first to examine the effects of physical exercise on 5-HTT and MAO-A promoter methylation, yet it did not yield significant results despite observing changes in 5-HTT expression levels. However, this does not rule out epigenetic regulation, as multiple mechanisms beyond DNA methylation are known to influence gene expression. Other epigenetic processes, such as histone modifications, non-coding RNAs, and chromatin remodelling, may also play a role in regulating 5-HTT expression in response to exercise. For example, physical exercise has been associated with global increases in histone H3 acetylation and although these changes are distributed across multiple genomic loci, they are accompanied by enhanced expression of genes involved in energy metabolism (McGee and Hargreaves 2011). Given the locus-specific nature of histone modifications, assessing histone H3 acetylation at regulatory regions of the 5-HTT gene could provide a more comprehensive view of exercise-induced epigenetic regulation than promoter CpG methylation alone. Nonetheless, the potential relevance of methylation changes at specific sites should not be disregarded. Rather, jointly assessing DNA methylation and hydroxymethylation (a dynamic and potentially reversible mark) together with methylation may be an important development for better capturing the stability and directionality of observed changes (Rustad et al. 2019), and for ultimately linking these epigenetic modifications to lifestyle or behavioural factors. Future research should explore these alternative pathways to better understand the molecular mechanisms underlying the observed changes.

## Conclusion

In conclusion, while our study demonstrates that HIIT can upregulate peripheral 5-HTT expression, this change does not directly correlate with reductions in impulsivity. This finding suggests that peripheral 5-HTT expression may not serve as a reliable proxy for central serotonergic activity related to impulsivity regulation. Future research should aim to elucidate the complex interactions within the serotonergic system and explore alternative peripheral biomarkers that more accurately reflect central processes.

## Supplementary Information

Below is the link to the electronic supplementary material.

Supplementary Material 1

## Data Availability

Data will be made available on reasonable request.

## Abbreviations

5-HIAA: 5-Hydroxyindoleacetic acid
5-HT: Serotonin (5-Hydroxytryptamine)
5-HTT: Serotonin transporter
5-HTTLPR: Serotonin transporter linked polymorphic region
ACTB: Actin beta
ANCOVA: Analysis of covariance
ANOVA: Analysis of variance
BC: Buffy coat
chr: Chromosome
CNS: Central nervous system
CpG: Cytosine-phosphate-guanine
Ct: Cycle threshold
(n)ERI: (non-)Emotion-related impulsivity
MAO-A: Monoamine oxidase A
MIQE: Minimum information for publication of quantitative real-time PCR experiments
MRT: Minus reverse transcriptase
MSRE: Methylation-sensitive restriction enzyme
NTC: Non-target control
PBS: Phosphate-buffered saline
RSP18: Ribosomal protein S18
RTqPCR: Reverse transcription quantitative polymerase chain reaction
STin2: Serotonin transporter intron 2 polymorphic region
Tm: Melting temperature (PCR/qPCR)
