# Supplementary material for: Eight weeks of high-intensity interval training increases peripheral serotonin transporter expression independently of promoter methylation changes
Source: Eur J Appl Physiol. 2026 Mar 14;126(7):3809–19. doi: 10.1007/s00421-026-06183-z (PMC13380567; doi:10.1007/s00421-026-06183-z)
Supplement: Supplementary file 1 — Supplementary Material 1 [file 421_2026_6183_MOESM1_ESM.docx]

**Supplementary Material**

**Eight Weeks of High-Intensity Interval Training Increases Peripheral Serotonin Transporter Expression Independently of Methylation Changes**

**F. Javelle** ^a^**, M. Ringleb** ^a, b, c^**, A. Schenk** ^d^**, W. Pulverer** ^e^**, W. Bloch** ^f^

Content:

- Design of DNA methylation assays and CpG sites coordinates
- Description of DNA methylation assessment
- Baseline Impulsivity levels per 5-HTTLPR phenotypes
- Baseline Correlations
- Effects of the intervention on Methylation levels

## Design of DNA methylation assays


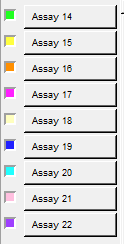

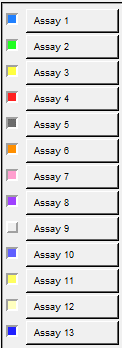

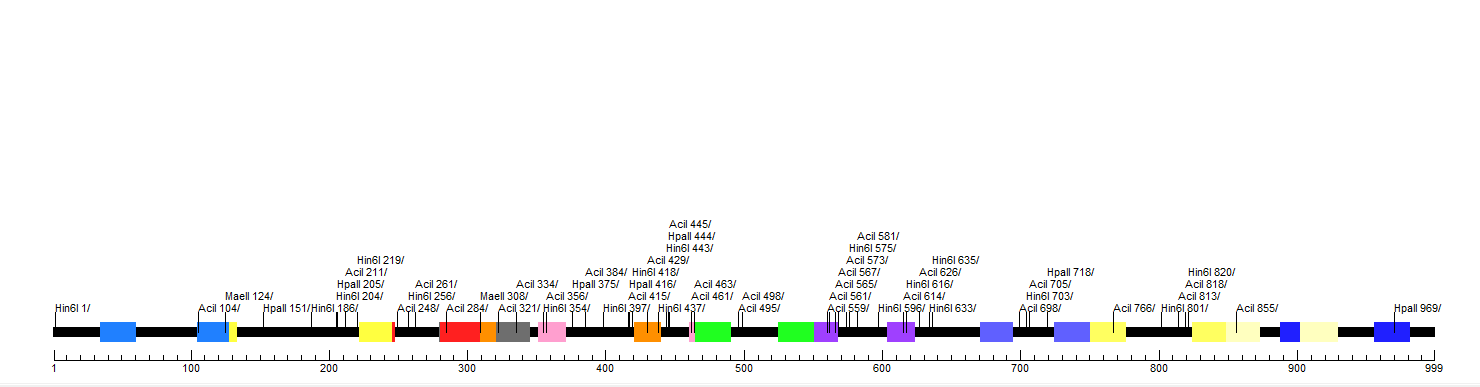

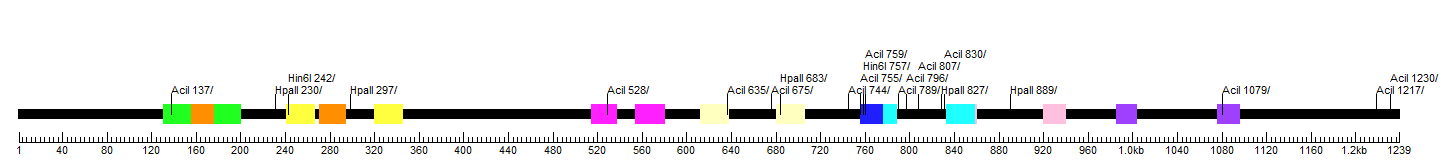


*Fig. A:* Design of the DNA Methylation Assays for SLC6A4


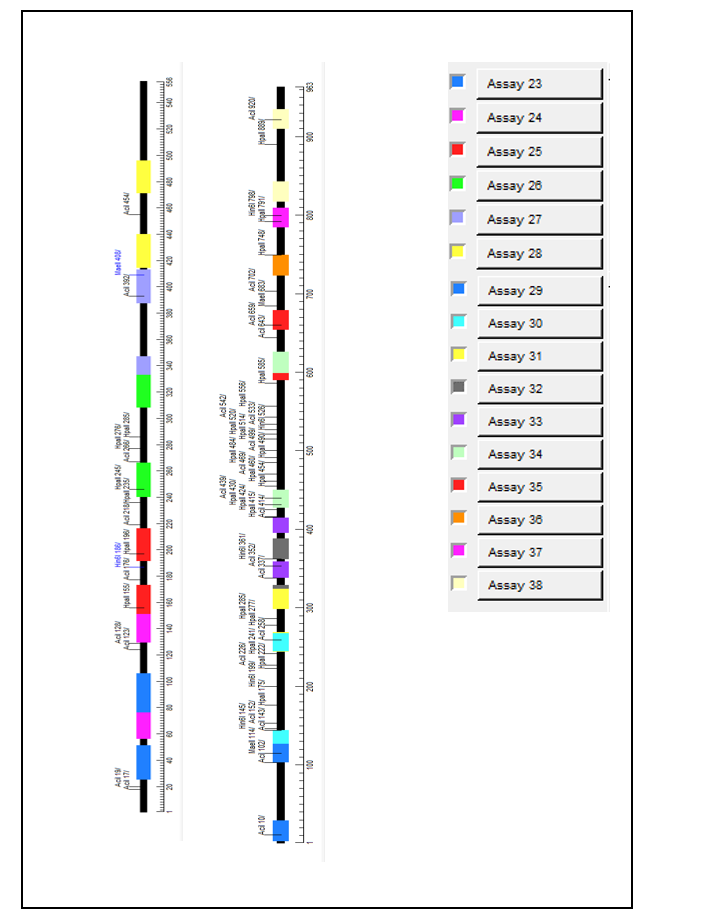


*Fig. B:* Design of the DNA Methylation Assays for MAO-A

The design of methylation assays for 5-HTT and MAO-A is presented in Fig. s A and B. Each assay contained one more CpG site (see Table 1). CpG sites repeated across multiple assays are colour-coded.

| **Gene** | **Assay** | **N** _unique_ **CpG, N** _repeated_ **CpG** | **Chromosome** | **Tested CpG Coordinates**  **_(according to the genomic Assembly GRCh38/Hg38)_** |  |
| --- | --- | --- | --- | --- | --- |
| SCL6A4 | 1 | 1, 0 | chr17 | 30,235,374 |  |
| SCL6A4 | 2 | 2, 0 | chr17 | 30,235,765  30,235,768 |  |
| SCL6A4 | 3 | 5, 1 | chr17 | 30,235,417  30,235,456  30,235,471  30,235,474  30,235,480  **30,235,489** |  |
| SCL6A4 | 4 | 2, 2 | chr17 | **30,235,489**  30,235,526  30,235,531  **30,235,554** |  |
| SCL6A4 | 5 | 0, 4 | chr17 | **30,235,554**  **30,235,578**  **30,235,591**  **30,235,604** |  |
| *SCL6A4* | *6* | *0, 12* | *chr17* | ***30,235,578***  ***30,235,591***  ***30,235,604***  ***30,235,624***  ***30,235,626***  ***30,235,641***  ***30,235,654***  ***30,235,667***  ***30,235,682***  ***30,235,685***  ***30,235,688***  ***30,235,699*** | *Excluded* |
| SCL6A4 | 7 | 5, 9 | chr17 | **30,235,624**  **30,235,626**  **30,235,641**  **30,235,654**  **30,235,667**  **30,235,682**  **30,235,685**  **30,235,688**  **30,235,699**  30,235,707  30,235,710  30,235,713  30,235,715  30,235,731 |  |
| SCL6A4 | 8 | 7, 3 | chr17 | 30,235,829  30,235,831  30,235,835  30,235,837  30,235,843  30,235,845  30,235,851  **30,235,866**  **30,235,884**  **30,235,886** |  |
| *SCL6A4* | *9* | *3, 3* | *chr17* | ***30,235,866***  ***30,235,884***  ***30,235,886***  *30,235,896*  *30,235,903*  *30,235,905* | *Excluded* |
| SCL6A4 | 10 | 4, 0 | chr17 | 30,235,968  30,235,973  30,235,975  30,235,984 |  |
| SCL6A4 | 11 | 5, 0 | chr17 | 30,236,036  30,236,071  30,236,083  30,236,088  30,236,090 |  |
| SCL6A4 | 12 | 1, 0 | chr17 | 30,236,125 |  |
| SCL6A4 | 13 | 1, 0 | chr17 | 30,236,235 |  |
| SCL6A4 | 14 | 1, 0 | chr17 | 30,236,406 |  |
| SCL6A4 | 15 | 1, 0 | chr17 | 30,236,562 |  |
| SCL6A4 | 16 | 2, 0 | chr17 | 30,236,495  30,236,511 |  |
| SCL6A4 | 17 | 2, 0 | chr17 | 30,236,797  30,236,841 |  |
| SCL6A4 | 18 | 3, 0 | chr17 | 30,236,904  30,236,944  30,236,984 |  |
| SCL6A4 | 19 | 4, 0 | chr17 | 30,237,013  30,237,024  30,237,026  30,237,028 |  |
| *SCL6A4* | *20* | *5, 0* | *chr17* | *30,237,058*  *30,237,065*  *30,237,076*  *30,237,092*  *30,237,099* | *Excluded* |
| *SCL6A4* | *21* | 1, 0 | *chr17* | 30,237,154 | *Excluded* |
| *SCL6A4* | *22* | 1, 0 | *chr17* | 30,237,348 | *Excluded* |
| MAO-A | 24 | 2, 0 | chrX: CpG island 1 | 43,656,101  43,656,106 |  |
| MAO-A | 25 | 4, 0 | chrX: CpG island 1 | 43,656,129  43,656,154  43,656,164  43,656,170 |  |
| MAO-A | 26 | 3, 0 | chrX: CpG island 1 | 43,656,244  43,656,250  43,656,259 |  |
| MAO-A | 27 | 2, 0 | chrX: CpG island 1 | 43,656,370  43,656,386 |  |
| *MAO-A* | *28* | *1, 0* | chrX: CpG island 1 | *43,656,432* | *Excluded* |
| MAO-A | 29 | 3, 0 | chrX: CpG island 2 | 43,654,693  43,654,785  43,654,797 |  |
| *MAO-A* | *30* | *8, 1* | chrX: CpG island 2 | *43,654,826*  *43,654,828*  *43,654,834*  *43,654,854*  *43,654,882*  *43,654,901*  *43,654,909*  *43,654,920*  ***43,654,941*** | *Excluded* |
| MAO-A | 31 | 2, 1 | chrX: CpG island 2 | **43,654,941**  43,654,956  43,654,964 |  |
| MAO-A | 32 | 1, 2 | chrX: CpG island 2 | 43,655,020  **43,655,035**  **43,655,044** |  |
| MAO-A | 33 | 0, 2 | chrX: CpG island 2 | **43,655,035**  **43,655,044** |  |
| MAO-A | 34 | 14, 0 | chrX: CpG island 2 | 43,655,122  43,655,132  43,655,139  43,655,152  43,655,163  43,655,169  43,655,182  43,655,193  43,655,199  43,655,209  43,655,216  43,655,225  43,655,235  43,655,263 |  |
| *MAO-A* | *35* | *0, 2* | chrX: CpG island 2 | ***43,655,326***  ***43,655,342*** | *Excluded* |
| MAO-A | 36 | 2, 3 | chrX: CpG island 2 | **43,655,326**  **43,655,342**  43,655,366  43,655,385  **43,655,427** |  |
| MAO-A | 37 | 2, 1 | chrX: CpG island 2 | **43,655,427**  43,655,470  43,655,481 |  |
| MAO-A | 38 | 2, 0 | chrX: CpG island 2 | 43,655,568  43,655,603 |  |

## Description of DNA methylation assessment

For the µ-fluidic DNA methylation readout on the Biomark system, digested and undigested DNA were subjected to multiplex preamplification for 22 cycles, containing primer pairs for all assays. The Biomark device allows up to 96 samples with up to 96 target regions to be analysed simultaneously, so that up to 9216 qPCR data points can be measured in a single run. For the present study, a smaller device was used, covering 48 samples and 48 target regions (=2304 reactions). For the preamplification step, a primer mixture was prepared consisting of the combined primer pairs and diluted to a final concentration of 200 nM per primer. For the preamplification reaction, 2.5 µl of TaqMan PreAmp Master Mix (Applied Biosystems, Foster City, California), 1.25 µl of the prepared 200 nM primer mix and 1.25 µl (25 ng) of the digested DNA were combined. The following PCR program was used for the preamplification of the targets of interest: 95 °C for 10 min, followed by 17 cycles of 95 °C for 15 s and 65 °C for 4 min. After preamplification, the reactions were diluted 1:5 with H2O. Two mixtures were prepared for DNA methylation readout of the samples: First, a sample mix containing 2 µl of the diluted sample from the preamplification, 0.25 µl of 20x EvaGreen (Biotium, California, USA), 0.25 µl of 20x DNA Binding Dye Sample Loading Reagent (Fluidigm, San Francisco, USA) and 2.5 µl of 2x TaqMan Gene Expression Master Mix (Applied Biosystems, Foster City, California). Secondly, an assay mix containing 2.5 µl of assay loading reagent (Fluidigm, San Francisco, USA), 0.25 µl of water, and 2.25 µl of pooled forward and reverse primers (20 µM per primer). The two mixtures were applied to Fluidigm's 48.48 GE Dynamic Array according to the manufacturer's protocol. The cycling conditions were as follows: Thermal mixing: 50 °C for 2 min, 70 °C for 30 min, 25 °C for 10 min; hot start: 95 °C for 15 min; followed by 35 cycles at 95 °C for 40 s, 65 °C for 40 s and 72 °C for 80 s; final elongation: 72 °C 7 min. Finally, a melting curve measurement was performed.

No preamplification was required for the readout on the Roche LightCycler, and the readout was performed in single reactions. A master mix with 20 ng DNA, 5µl 2x TaqMan Gene Expression Master Mix (Applied Biosystem, Foster City, California), 0.25µl 20x EvaGreen (Biotium, California, USA) and PCR water was made up to 10µl. The PCR programme consisted of a hot start at 95 °C for 15 min, followed by 45 cycles at 95 °C for 40 s, 65 °C for 40 s, and 72 °C for 80 s, and concluded with a melting curve measurement.

## Baseline Impulsivity levels per 5-HTTLPR phenotypes

A

**
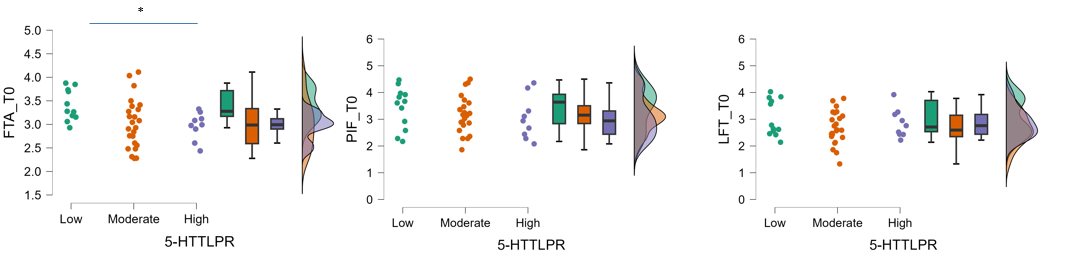
**

C

B

Fig. C: Baseline A) Feelings Trigger Action (FTA), B) Pervasive Influence of Feelings (PIF) and C) Lack of Follow-Through (LFT) levels per 5-HTTLPR phenotypes. * : p<.050; **: p<.010; ***: p<.001.

Table A: 5-HTT promoter methylation levels between 5-HTTLPR transcriptional activity phenotypes. Note: Higher values indicate lower levels of methylation in the assay.

| Assay | n | 5-HTT_Low_ | 5-HTT_Moderate_ | 5-HTT_High_ | *Group difference _(p)_* | *Log_2_*  *(Low/Mod.)*  *Fold changes* | *Log_2_*  *(Low/High)*  *Fold changes* |
| --- | --- | --- | --- | --- | --- | --- | --- |
| 1 | 43 | 1.41 ± 0.74 | 1.33 ± 1.08 | 1.31 ± 8.63 | .970 | 0.08 | 0.11 |
| 2 | 44 | 1.93 ± 1.14 | 4.57 ± 7.22 | 3.95 ± 7.43 | .524 | -1.28 | -1.03 |
| 3 | 45 | 21.73 ± 10.26 | 18.58 ± 11.87 | 19.14 ± 11.07 | .578 | 0.23 | 0.18 |
| 4 | 45 | 20.22 ± 9.43 | 18.66 ± 10.93 | 22.92 ± 8.01 | .557 | 0.12 | -0.18 |
| 5 | 35 | 1.97 ± 1.32 | 4.53 ± 7.24 | 1.82 ± 1.56 | .295 | -1.20 | 0.11 |
| **7** | **44** | **2.18 ± 1.40** | **3.09 ± 1.55** | **1.48 ± 1.51** | **.028** | **-0.50** | **0.55** |
| 8 | 44 | 6.22 ± 2.06 | 6.42 ± 1.93 | 5.95 ± 2.66 | .849 | -0.05 | 0.06 |
| 10 | 45 | 5.46 ± 1.86 | 5.25 ± 1.58 | 5.40 ± 2.00 | .939 | 0.06 | 0.02 |
| 11 | 30 | 23.74 ± 7.74 | 19.93 ± 10.52 | 18.57 ± 10.67 | .566 | 0.25 | 0.35 |
| 12 | 42 | 0.43 ± 0.34 | 1.67 ± 5.49 | 0.23 ± 0.27 | .608 | -1.95 | 0.90 |
| **13** | **45** | **2.15 ± 0.34** | **2.05 ± 0.90** | **1.20 ± 0.76** | **.006** | **0.07** | **0.84** |
| 14 | 45 | 0.18 ± 0.24 | 0.29 ± 0.46 | 0.39 ± 0.09 | .231 | -0.69 | -1.12 |
| 15 | 45 | 0.09 ± 0.14 | 0.23 ± 0.45 | 0.13 ± 0.26 | .544 | -1.35 | -0.53 |
| 16 | 44 | 0.02 ± 0.06 | 0.14 ± 0.32 | 0.08 ± 0.15 | .467 | -2.81 | -2.00 |
| 17 | 44 | 1.49 ± 0.44 | 1.59 ± 0.86 | 2.29 ± 2.78 | .383 | -0.09 | -0.62 |
| 18 | 44 | 5.94 ± 7.77 | 3.28 ± 1.47 | 2.74 ± 1.52 | .136 | 0.86 | 1.12 |
| 19 | 45 | 3.08 ± 1.74 | 2.90 ± 1.39 | 2.69 ± 1.25 | .834 | 0.09 | 0.20 |


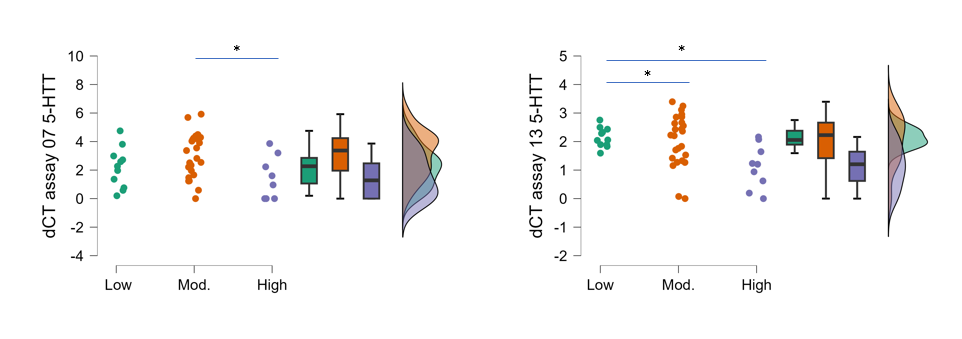


Fig. D: 5-HTT promoter Methylation levels in each 5-HTTLPR phenotypic group. A) Presents the assay 07 and B) presents the assay 13. * : p<.050; **: p<.010; ***: p<.001.

B

A

Table B: MAO-A promoter methylation levels between MAO-A transcriptional activity phenotypes. Note: Higher values indicate lower levels of methylation in the assay.

| Assay | n | MAO-A_Low_ | MAO-A_High_ | *Group difference _(p)_* | *Log_2_*  *(Low/High)*  *Fold changes* |
| --- | --- | --- | --- | --- | --- |
| 24 | 45 | 2.72 ± 8.05 | 0.05 ± 0.80 | .148 | 5.77 |
| 25 | 45 | 6.14 ± 8.68 | 7.70 ± 9.89 | .576 | -0.33 |
| **26** | **45** | **4.49 ± 8.01** | **11.41 ± 11.95** | **.025** | **-1.35** |
| 27 | 45 | 2.37 ± 5.80 | 1.32 ± 1.10 | .430 | 0.84 |
| 29 | 43 | 5.66 ± 8.44 | 9.00 ± 2.65 | .280 | -0.67 |
| 31 | 41 | 12.81 ± 12.52 | 18.14 ± 11.94 | .176 | -0.50 |
| 32 | 45 | 9.22 ± 10.60 | 12.45 ± 11.87 | .341 | -0.43 |
| 33 | 45 | 1.48 ± 0.98 | 2.77 ± 6.33 | .320 | -0.90 |
| 34 | 40 | 9.60 ± 11.35 | 12.10 ± 12.39 | .509 | -0.33 |
| 36 | 44 | 6.53 ± 10.64 | 10.69 ± 13.00 | .250 | -0.71 |
| **37** | **43** | **2.14 ± 1.94** | **4.00 ± 3.11** | **.021** | **-0.90** |
| 38 | 45 | 3.42 ± 5.65 | 7.41 ± 9.66 | .091 | -1.12 |


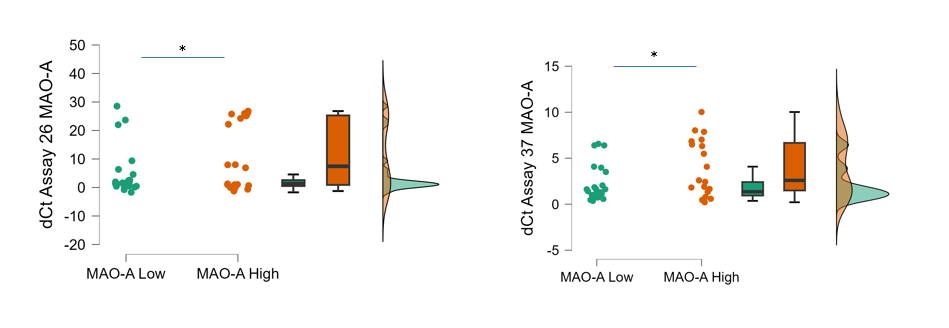


A

B

Fig. E: MAO-A promoter methylation levels in each MAO-A VNTR phenotypic group. A) Presents the assay 26 and B) presents the assay 37. * : p<.050; **: p<.010; ***: p<.001.

##
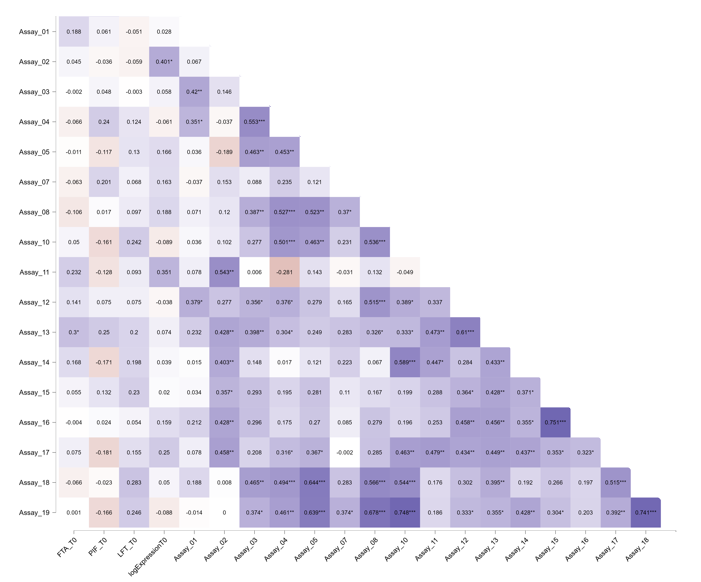
Baseline Correlations

Fig. F: Heatmap of Baseline Spearman correlations for the different methylation assays, impulsivity levels and 5-HTT expression. * : p<.050; **: p<.010; ***: p<.001.


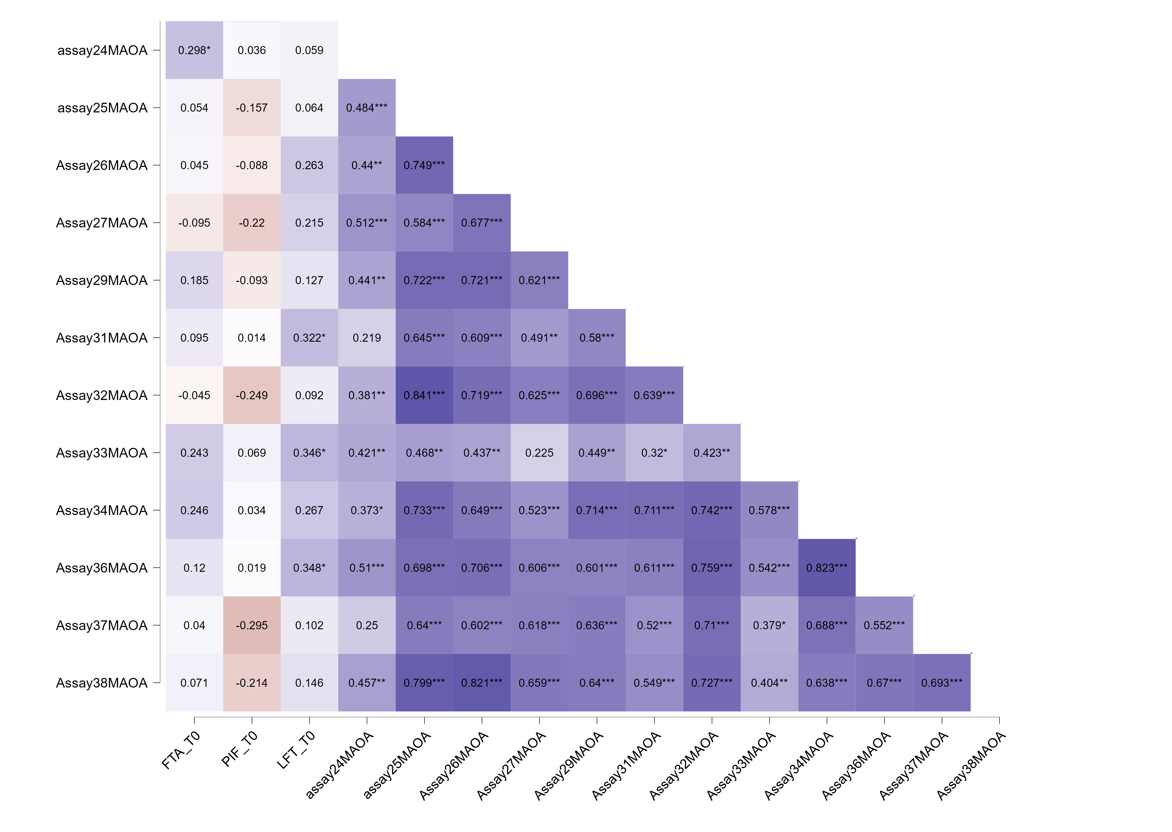


Fig. G: Heatmap of Baseline Spearman correlations for the different MAO-A methylation assays, impulsivity levels. * : p<.050; **: p<.010; ***: p<.001.

## Effects of the intervention of Methylation levels

Table D: Effect of the intervention on 5-HTT and MAO-A promoter methylation levels. Gender controlled ANCOVAs of the ddCt scores. Notes: A positive number refers to an increase in methylation, while a negative number refers to a decrease in methylation.

| Assay | Gene | n | HIIT _(mean ±SD)_ | Stretching _(mean ±SD)_ | *Group difference _(p)_* |
| --- | --- | --- | --- | --- | --- |
| 1 | 5-HTT | 42 | 0.15 ± 1.18 | -0.57 ± 2.50 | .203 |
| 2 | 5-HTT | 43 | 1.87 ± 6.77 | 0.95 ± 6.16 | .656 |
| 3 | 5-HTT | 45 | -2.95 ± 14.13 | - 3.11 ± 18.35 | .897 |
| 4 | 5-HTT | 44 | -1.01 ± 15.43 | -3.11 ± 14.27 | .594 |
| 5 | 5-HTT | 22 | -2.22 ± 9.30 | -1.83 ± 11.06 | .900 |
| 7 | 5-HTT | 44 | -1.92 ± 3.47 | -1.80 ± 3.74 | .992 |
| 8 | 5-HTT | 43 | -1.92 ± 2.67 | -2.19 ± 2.36 | .804 |
| 10 | 5-HTT | 45 | -0.37 ± 3.22 | -0.07 ± 2.95 | .738 |
| 11 | 5-HTT | 29 | 8.63 ± 20.03 | 3.07 ± 15.34 | .143 |
| 12 | 5-HTT | 42 | -0.22 ± 0.59 | -0.39 ± 10.14 | .916 |
| 13 | 5-HTT | 44 | -0.34 ± 1.04 | -0.85 ± 2.81 | .436 |
| 14 | 5-HTT | 45 | 0.20 ± 0.50 | 0.05 ± 0.28 | .267 |
| 15 | 5-HTT | 45 | -0.27 ± 0.80 | -0.93 ± 3.08 | .365 |
| 16 | 5-HTT | 44 | -0.18 ± 0.50 | -0.90 ± 3.21 | .281 |
| 17 | 5-HTT | 44 | -2.23 ± 7.00 | 0.17 ± 1.95 | .083 |
| 18 | 5-HTT | 44 | -1.67 ± 2.04 | -4.09 ± 10.76 | .234 |
| 19 | 5-HTT | 44 | -1.48 ± 1.55 | -1.71 ± 1.66 | .757 |
| 24 | MAO-A | 44 | 1.14 ± 10.40 | -1.76 ± 7.01 | .295 |
| 25 | MAO-A | 45 | -2.72 ± 10.80 | -5.33 ± 13.07 | .188 |
| 26 | MAO-A | 45 | -2.49 ± 7.91 | - 2.64 ± 13.04 | .669 |
| 27 | MAO-A | 45 | -0.97 ± 5.55 | -1.58 ± 7.01 | .230 |
| 29 | MAO-A | 42 | 1.31 ± 7.41 | 3.83 ± 13.89 | .295 |
| 31 | MAO-A | 40 | 2.56 ± 8.81 | 4.68 ± 13.74 | .754 |
| 32 | MAO-A | 43 | -1.76 ± 8.47 | -1.53 ± 13.02 | .895 |
| 33 | MAO-A | 45 | -0.42 ± 7.64 | -1.03 ± 2.87 | .743 |
| 34 | MAO-A | 39 | -0.03 ± 7.56 | -4.68 ± 8.63 | .100 |
| 36 | MAO-A | 44 | -2.82 ± 10.53 | -3.17 ± 8.48 | .679 |
| 37 | MAO-A | 43 | -0.61 ± 2.35 | -1.18 ± 1.65 | .142 |
| **38** | **MAO-A** | **44** | **1.41 ± 8.26** | **-5.74 ± 10.77** | **.018** |


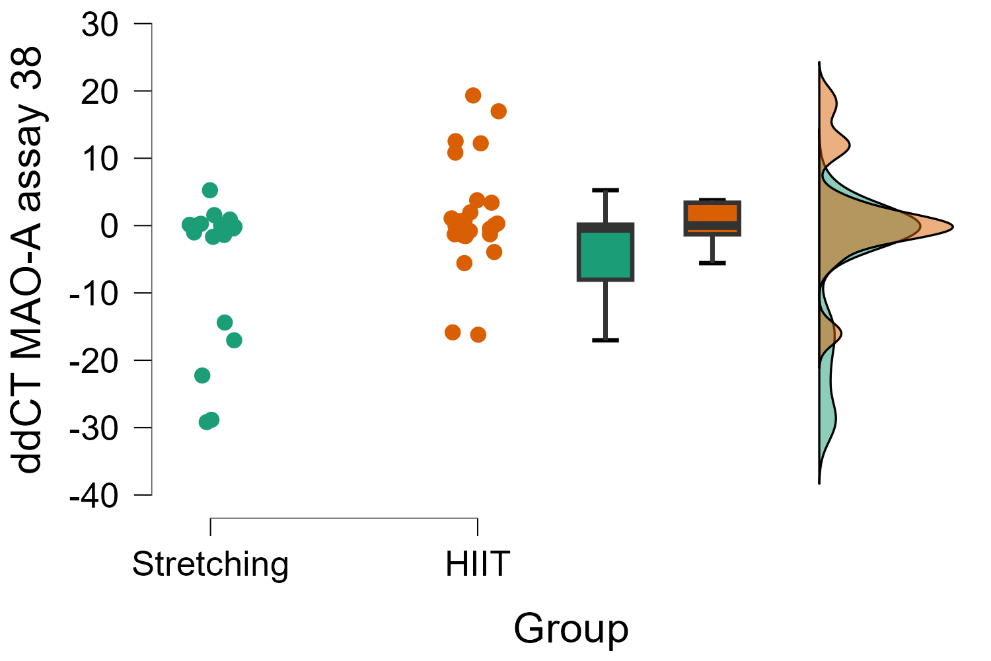


Fig. H: MAO-methylation levels on assay 38 from T0 to T8 between stretching and HIIT group. * : p<.050; **: p<.010; ***: p<.001.


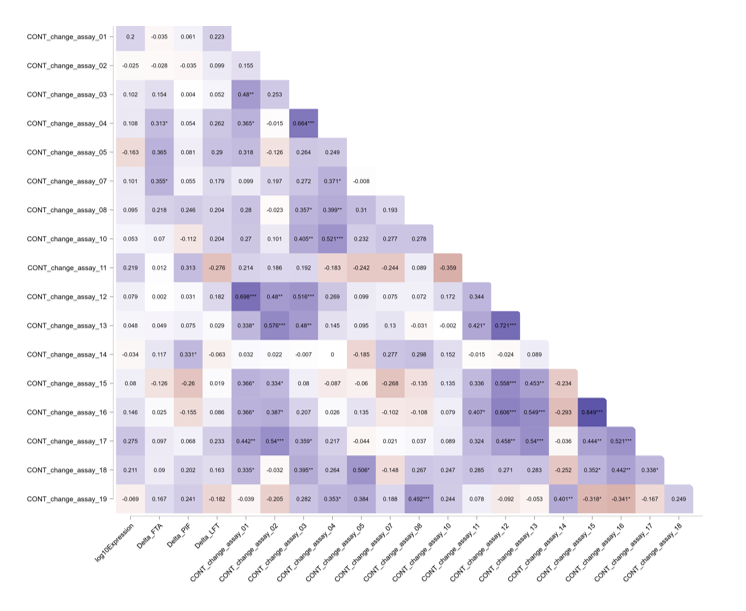


Fig. I: Heatmap of delta Spearman correlations for the different 5-HTT methylation assays, impulsivity levels and 5-HTT expression. * : p<.050; **: p<.010; ***: p<.001.


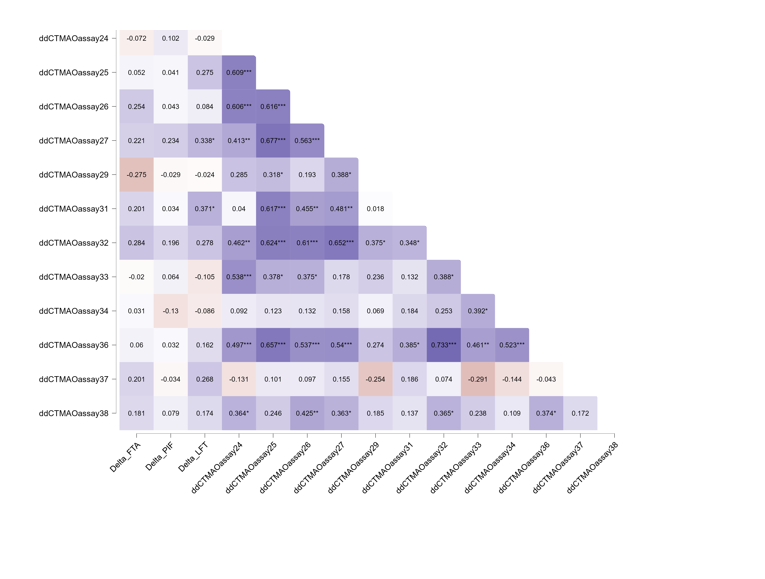


Fig. J: Heatmap of delta Spearman correlations for the different MAO-A methylation assays and impulsivity levels. * : p<.050; **: p<.010; ***: p<.001.
